# The Book World of Medicine and Science

**Published:** 1895-08-17

**Authors:** 


### The Book World of Medicine and Science.

Burdett's Hospital and Charities Annual, 1895. By Henry C. Burdett. (London: Scientific Press. 1895. Pp. 834, price 5s.)

Among the various annuals which appear year by year devoted each to its own individual interest "Burdett's Hospital and Charities Annual" stands unique, and in some sense apart, seeing that it is not to one class alone that it appeals, but to the whole of that large public by whom the charities of England are supported, as well as to that large section of the community by whom, whether as governors, visitors, members of committees, secretaries, or other officials, these charities are managed and maintained in being.

The magnitude of what one may call the business of charity is far greater than many people would imagine. "London contains a greater number of charities of all kinds than any other city in the world, and the combined revenues of these charities is so great as to stagger the uninitiated when brought face to face with the total for the first time. The income of the greater charities of every kind which have their headquarters in London amounts to upwards of £7,000,000 per annum, a sum which exceeds the total revenue of all but three British colonies—New South Wales, Victoria, and Canada—at the present time." It is interesting, and not altogether satisfactory, to find how large a proportion of this goes in foreign missions, and no doubt there are many who grudge the large expenditure of money and energy on those outside our borders, whilst so many are uncared for within.

Great emphasis is laid on the importance of a complete audit being enforced in the case of all charities, especially those which may be spoken of as general charities, the objects of which are not always too well defined. Some of them even employ amateur auditors, but even when chartered accountants are asked to undertake the task, it is to be feared that very often their examination is perfunctory and superficial. "If the true function of an audit is to make things go smoothly, they perform their function in an admirable manner. But the true function of the auditor is to check extravagance, to detect dishonesty, to call attention to useless or improper expenditure. How often is this done by the friendly accountant?" Words such as these, written by one who is well known as an expert in hospital management, should not be lightly put aside by the managers of charities which depend absolutely for their success and usefulness upon the confidence of the public, for this is a critical and fault-finding generation, and if confidence is to be maintained all must be open and above board.

The author is stringent in his criticism of what is commonly spoken of as "the one man show." "Once more," he says, "we must repeat the caution—beware of professional philanthropists. Societies founded or controlled by one man, who lives on his share of the subscriptions, are always to be regarded with suspicion. He may parade a list of names, more or less well known, as a committee, but it is too often a committee in name only, and the man himself has the entire management of the institution and its funds."

In regard to orphanages, &c., it seems "countless organisations, embracing the care of from six to 6,000 children, gather in a yearly revenue of over £1,000,000 sterling, and some 54,000 children are rescued by this means from want and vicious surroundings." This is a grand thing, of which the subscribers to these institutions may well be proud. At the same time "the drawbacks to institution life have long been recognised by the experienced. . . . . Notwithstanding all improvements, the life of the institution remains an unnatural one. The child is inevitably excluded from any experience of the duties, dangers, and responsibilities of family life, and passes, when the time of seclusion is over, to freedom and temptation for which the laborious training of years has supplied no practical preparation." This especially applies to those institutions where children are admitted in infancy and remain till the age of seventeen or eighteen. "Three or four years are found amply sufficient for establishing orderly habits and imparting industrial training to serve as a basis for future employment," and Mr. Burdett compares the effect of such a residence to that produced in a higher walk in life by going to a good boarding school. For little children, however, the institution life can never be anything but an evil; for them the boarding-out system is the best alternative.

To turn, however, to the subject of hospitals, we find an enormous mass of information, together with much criticism, which, although evidently given in a friendly and sympathetic spirit, is none the less stringent where evils and abuses are concerned.

One of the most satisfactory features in the survey which is here given of hospital finance is to be found in the statement that much as charity of all sorts may be, and as a fact is, influenced and reduced in volume by bad times, still, as regards the hospitals, charity is not *in extremis*. The figures given, which are tabulated in a most interesting and painstaking manner, show that, with a few notable exceptions, the voluntary hospitals are, financially speaking, in a surprisingly satisfactory condition. This generation ought evidently, however, to do more than it does for its hospitals, unless it is willing to admit that it is living on the good works of its ancestors. It certainly will come as a surprise to many to learn that "of every sovereign expended by the hospitals and dispensaries of London (excluding St. Thomas's and St. Bartholomew's, which are largely endowed) only nine shillings are received from the living, *i.e.*, the present inhabitants of London, for whose benefit the hospitals chiefly exist"; and it may well be asked what self-respect there can be in a population which is content to leave the hospitals seriously crippled for funds, while the present generation only contributes about one-third, or in any case less than one-half, of the value received from the hospitals in medical relief?

The tables giving detailed analyses of the expenditure of the various hospitals in the country must have cost an immense amount of labour in their preparation, and if read carefully are full of instruction to those responsible for the management of these institutions. Separate tables are devoted to the different classes of hospitals, both London, Provincial, Scotch, and Irish; and some statistics of the same character, but not so complete, are given regarding the hospitals in the United States.

Many things have to be borne in mind in comparing the expenditure of different hospitals, and it is important not to draw hasty inferences; nevertheless it is impossible not to be struck with the great differences between the expenditure of the various hospitals of the same class, on items coming under the same heads; and one cannot but believe that a careful study by secretaries and committee-men of the tables given in this work would in many instances give the clue by which large economies might be initiated. As is well known, Mr. Burdett has devoted a large amount of study, not only to the structure of hospitals for the sick, but to the general principles on which they should be managed, and we would direct special attention to the remarks, in this edition, upon the various interesting questions which are now or soon will be before the public, such as remuneration of the staff, the acceptance of contributions from the patients for the support of the hospitals, and the wide question of the constantly-growing expense of hospital treatment.

"Burdett's Hospital and Charities Annual" has become a standard work of reference, and we need do no more than direct attention to its issue for 1895. At the same time it is impossible to withhold a word of praise for the beauty of its typography, and the admirable finish with which the volume is got up.

